# Fluvastatin suppresses the proliferation, invasion, and migration and promotes the apoptosis of endometrial cancer cells by upregulating Sirtuin 6 (SIRT6)

**DOI:** 10.1080/21655979.2021.2009415

**Published:** 2021-12-18

**Authors:** Yu Cai, Feng Zhao

**Affiliations:** a Gynecology Department, The Third People’s Hospital of Da Lian, Da Lian, China; b Obstetrics and Gynecology Department, Hankou Hospital, Wuhan, Hubei, China

**Keywords:** Fluvastatin, endometrial cancer, SIRT6

## Abstract

Fluvastatin, the first fully synthesized 3-Hydroxy-3-methylglutaryl coenzyme A (HMG-CoA) reductase (HMGCR) inhibitor, has been reported to inhibit the development and metastasis of multiple cancers. The present study aimed to explore the effects of fluvastatin on endometrial cancer (EC) as well as reveal its potential mechanism. After exposure to fluvastatin, the cell viability, proliferation, migration, and invasion of EC cells were measured by Cell Counting Kit-8 (CCK-8), 5-ethynyl-2ʹ-deoxyuridine (EDU), wound healing, and invasion assays, respectively. The apoptosis and its related proteins of fluvastatin-treated EC cells were detected by TUNEL and Western blot, separately. In order to figure out the effects of SIRT6 silence on EC cells, a series of cellular activities were performed again. Fluvastatin suppressed the proliferation, migration, and invasion of EC cells, but induced the apoptosis. The expression of SIRT6 was elevated in EC cells upon fluvastatin exposure. After silencing SIRT6 in fluvastatin-treated EC cells, the proliferation, migration, and invasion were promoted whereas the apoptosis was decreased. To sum up, this study firstly evidenced that fluvastatin suppresses the proliferation, invasion, and migration and promotes the apoptosis of endometrial cancer cells by regulating SIRT6 expression.

## Introduction

Endometrial cancer (EC) featuring rapidly increasing incidence is recognized as the fourth most common cancer among women in developed countries [1]. Risk factors are associated with excessive unopposed exposure of the endometrium to estrogen, including early menarche, late menopause, tamoxifen therapy, infertility, or failure to ovulate, and polycystic ovary syndrome [2]. A widely accepted perception is that EC is a disease mainly threating the health of older women [3]. Most women with EC, if diagnosed at the early stage, can obtain a good prognosis accompanied by a five-year survival rate that reaches as high as 95% [1]. However, it is estimated that the number of women diagnosed with this disease by 2030 will be two times bigger than this year [4]. Therefore, it is imperative to comprehensively understand EC pathogenesis and find more potent therapies for EC.

Fluvastatin is the first fully synthesized HMGCR inhibitor, which has been reported to suppress the development and metastasis of cancer [5,6]. A recent study showed that fluvastatin can effectively prevent bone metastasis of lung adenocarcinoma in nude mice [7]. In addition, fluvastatin can inhibit the proliferation and induce apoptosis of non-small cell lung cancer [8]. It was also testified that fluvastatin promotes the death of breast cancer cells, but the effects of fluvastatin on EC remain unknown [9]. Existing studies have shown that fluvastatin can activate the expression of SIRT6, and SIRT6 overexpression can induce apoptosis by inhibiting survivin expression [10,11]. We assume that fluvastatin possess an anti-tumor potential in EC by SIRT6. Therefore, we further explore whether fluvastatin can activate the expression of SIRT6 and affect the proliferation, invasion, migration as well as apoptosis of endometrial cancer cells, which may shed light on the role of fluvastatin in EC and reveal its underlying mechanism.

## Materials and methods

### Cell culture and treatment

Fluvastatin was purchased from wako (Osaka, Japan). Human EC cell lines including RL95-2 and KLE were purchased from the Cell Bank of Chinese Academy of Medical Sciences (Shanghai, China). Cells were maintained at 37°C with 5% CO_2_ and cultivated in DMEM containing 10% FBS. 48 h later, fluvastatin with different concentrations (5, 10, 15, 20 μm) were employed to incubate cells for 72 h for following experiments.

### RNA interference

shRNA against SIRT6 was used to knock down SIRT6 expression, and sequences of sh SIRT6 (5ʹ-CCGGTGGAAGAATGTGCCAAGTGTACTCGAGTACACTTGGCACATTCTTCCATTTTTG-3ʹ) were designed by Genomics Co., Beijing, People’s Republic of China. Transfection was performed by Lipofectamine™ 2000 transfection reagent (Invitrogen; Thermo Fisher Scientific, Inc.) based on the suggestions provided by the manufacturer. After transfection of 48 h, cells were used to perform further experiment.

### CCK-8 assay

For the detection of cell viability, cells at the concentration of 4 × 10^3^ cells per well were placed into 96-well plates and cultured for 24 h. Following the cell culture, 10 µl of Cell Counting Kit-8 solution was added into each well to incubate the cells, and the absorbance at 450 nm was assessed by a microplate auto-reader (Thermo Fisher Scientific).

### EDU assay

EC cells (4 × 10^3^ cells/well) that had been inoculated into 96-well plates were cultured for 48 h, after which was the replacement of the original medium by medium containing 50 μm EDU. 2 h post incubation, cells were fixed with 4% formaldehyde for 30 min and permeabilized with 0.5% Triton X-100 for 15 min. Afterward, cells were counterstained with anti-EdU reagents for 30 min. Following the incubation with 100 μL DAPI for 30 min, a fluorescence microscope (Olympus, Tokyo, Japan) was used to assess the proliferation.

### Wound healing

Cells after transfection were trypsinized and plated in the inserts of the 96-well plates, and a pipette tip was used to create a wound on the monolayer of transfected cells. Fresh medium was placed into the plates immediately to discard the floating cells, and the scratch as well as surrounding cells were recorded. Then, photographs were obtained at 24 h to determine the wound closure using microscope (Olympus, Tokyo, Japan).

### Transwell

Cell invasion was tested in chamber of 8-mm Transwell inserts with Matrigel. EC cells were added to the upper chamber of each insert in serum-free medium, and serum medium was utilized in the lower chamber as the attractant. Migrated cells were fixed using 4% paraformaldehyde for 0.5 h and stained by crystal violet for 20 min. The migrated cells were counted and visualized by an inverted microscope (Olympus, Tokyo, Japan).

### Western blot

Total protein was extracted from cell lysates after cells were harvested and centrifuged at 4°C and 16,000 × g for 20 min. Protein concentrations were measured by BCA method (Thermo Fisher Scientific, Inc.). Subsequently, proteins were separated via 10% SDS-PAGE for 2 h and transferred onto PVDF membranes by means of a wet transfer electrophoresis tank (Bio-Rad Laboratories, Inc.) for 2 h. Sealed by 5% skim milk for 1 h at room temperature, the membranes were then incubated overnight at 4°C with the following primary antibodies: anti-SIRT6 (1:2,000; cat. no. ab191385; Abcam); anti-cleaved-caspase-3 (Asp175; 1:2,000; cat. no. 9664; Cell Signaling Technology, Inc.); anti-p53 (1:10,000; cat. no. ab32389; Abcam); anti-MMP12 (1:1,000; cat. no. ab52897; Abcam); anti-MMP9 (1:1,000; cat. no. Ab76003; Abcam); anti-GAPDH (1:2,000; cat. no. ab181602; Abcam). After being washed with PBS for twice, the membranes were further incubated with horseradish peroxidase-conjugated secondary antibody (1:2,000; cat. no. ab6721; Abcam). Visualization of bands was conducted by electrochemiluminescence substrate (Thermo Fisher Scientific, Inc.).

### RT-qPCR

Total RNA from the harvested EC cells was extracted using TRIzol Reagent (Thermo Fisher Scientific) and then the RNA was reversed to cDNA by means of a HiFiScript cDNA Synthesis kit (CwBio, Beijing, China) in accordance with the manufacturer’s protocol. RT-qPCR was performed by QuantiTect SYBR-Green PCR kit (both from Takara Biotechnology Co., Ltd., Dalian, China) on an ABI 7500 Fast System Thermocycler (Thermo Fisher Scientific, Inc.). 2^−ΔΔCT^ method was applied to calculate the quantitative results and the relative expression level of each gene was normalized against GAPDH [12].

### TUNEL

The cell apoptosis was determined by a TUNEL fluorescence kit (Hoffman-La Roche Ltd.), and DAPI (1:5,000; Beyotime, Beijing, People’s Republic of China) was used to stain the nuclei. The apoptosis of EC cells was evaluated by calculating the number of TUNEL-positive cells under a Laser Scanning Confocal Microscope.

### Statistical analysis

The acquired data were presented as mean ± SD. Statistical analysis was processed by GraphPad Prism 5.0 (GraphPad Software, La Jolla, CA, USA). Comparisons among groups were conducted by one-way ANOVA, followed by Tukey’s post hoc test. A value of P < 0.05 means significant difference.

## Results

### Fluvastatin suppresses the proliferation, migration, and invasion of EC cells

**Figure 1.**
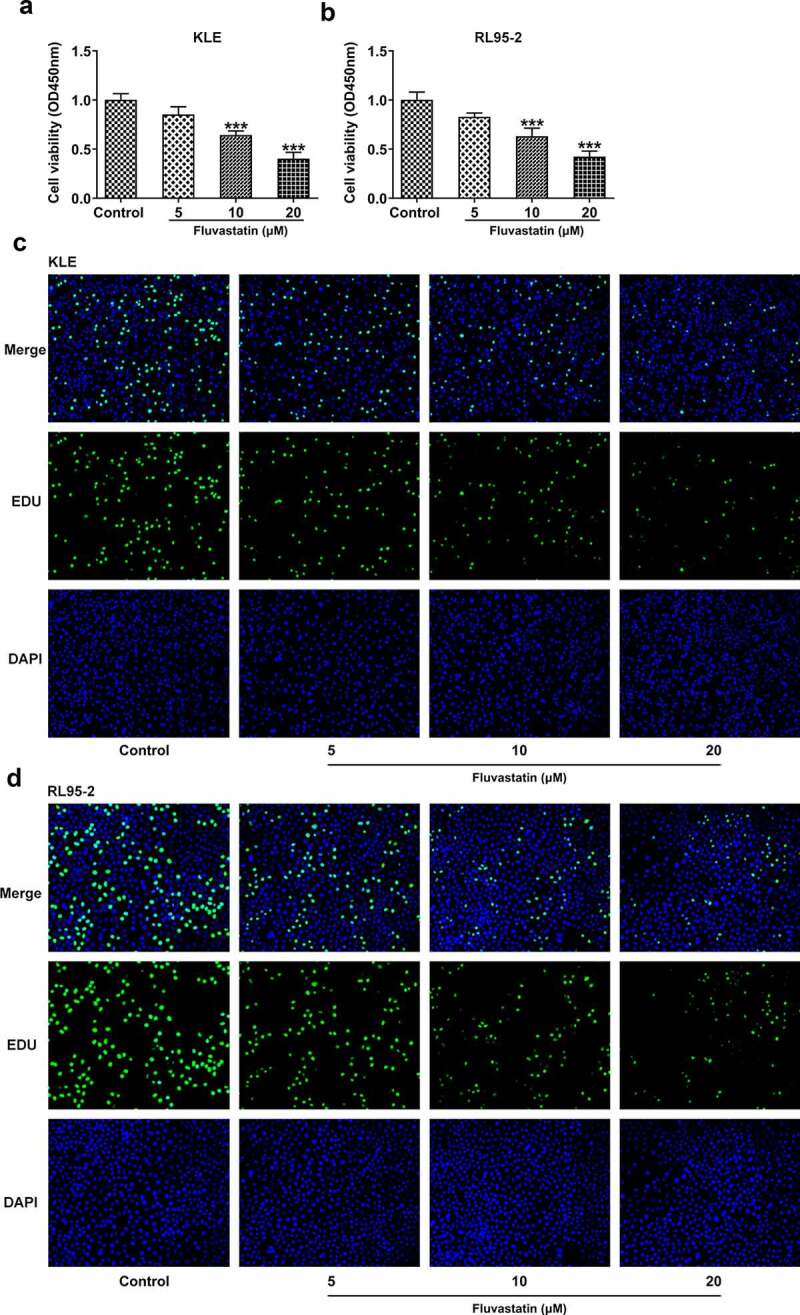
Fluvastatin suppresses the proliferation of EC cells. (a-b) The cell viability of EC cells exposed to fluvastatin. (c-d) The proliferation of EC cells exposed to fluvastatin. *** P < 0.001 versus control

**Figure 2.**
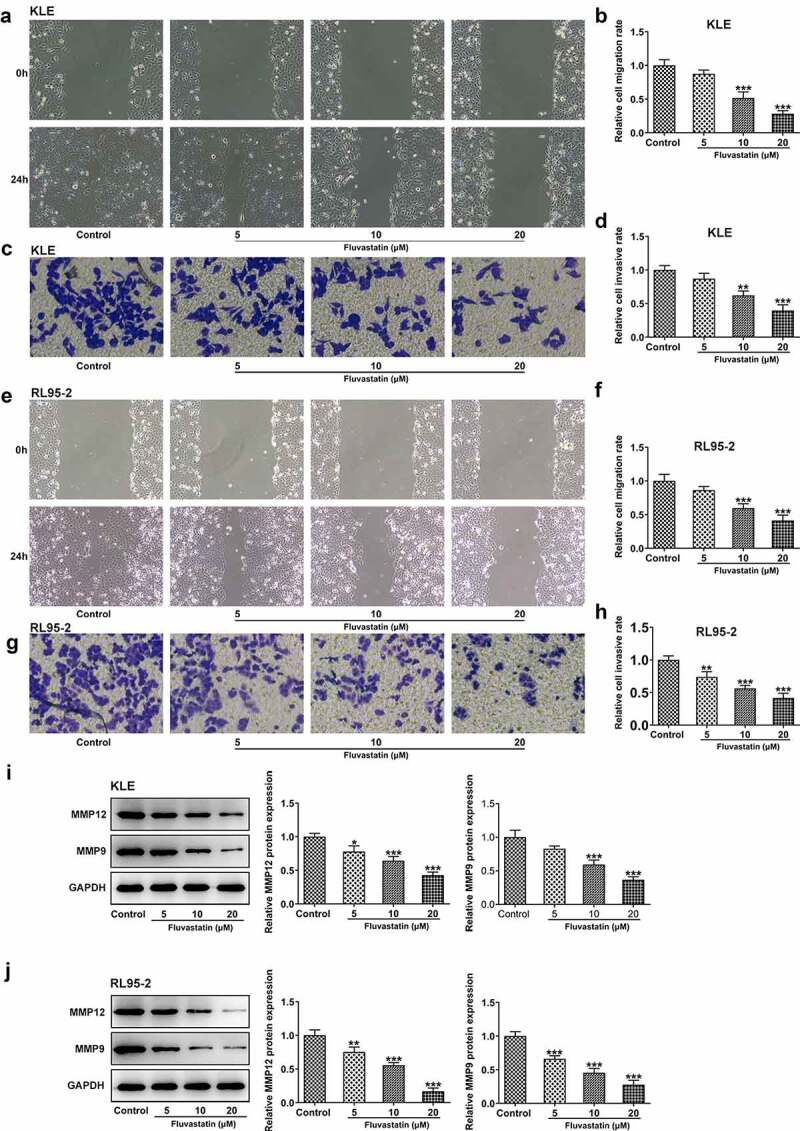
Fluvastatin suppresses the migration and invasion of EC cells. (a-b) The migration, (c-d) invasion, (e-f) migration, (g-h) invasion, (i-j) MMP12 and MMP9 expressions in EC cells exposed to fluvastatin. *P < 0.05, **P < 0.01, *** P < 0.001 versus control

To explore the impacts of fluvastatin on the functions of EC cells, we firstly measured the proliferation of fluvastatin-treated KLE and RL95-2 cells by CCK-8 and EDU assays. As exhibited in Figure 1a-d, the viability and proliferation of KLE and RL95-2 cells were decreased by fluvastatin in a concentration-dependent manner. In addition, results in Figure 2a-h demonstrated the favorable effects of fluvastatin on suppressing the migration and invasion of EC cells. The downregulated expressions of MMP12 and MMP9 in fluvastatin-treated KLE and RL95-2 cells also suggested the potency of fluvastatin in inhibiting the malignant progression of EC cells (Figure 2i-j).

### Fluvastatin induces the apoptosis of EC cells

**Figure 3.**
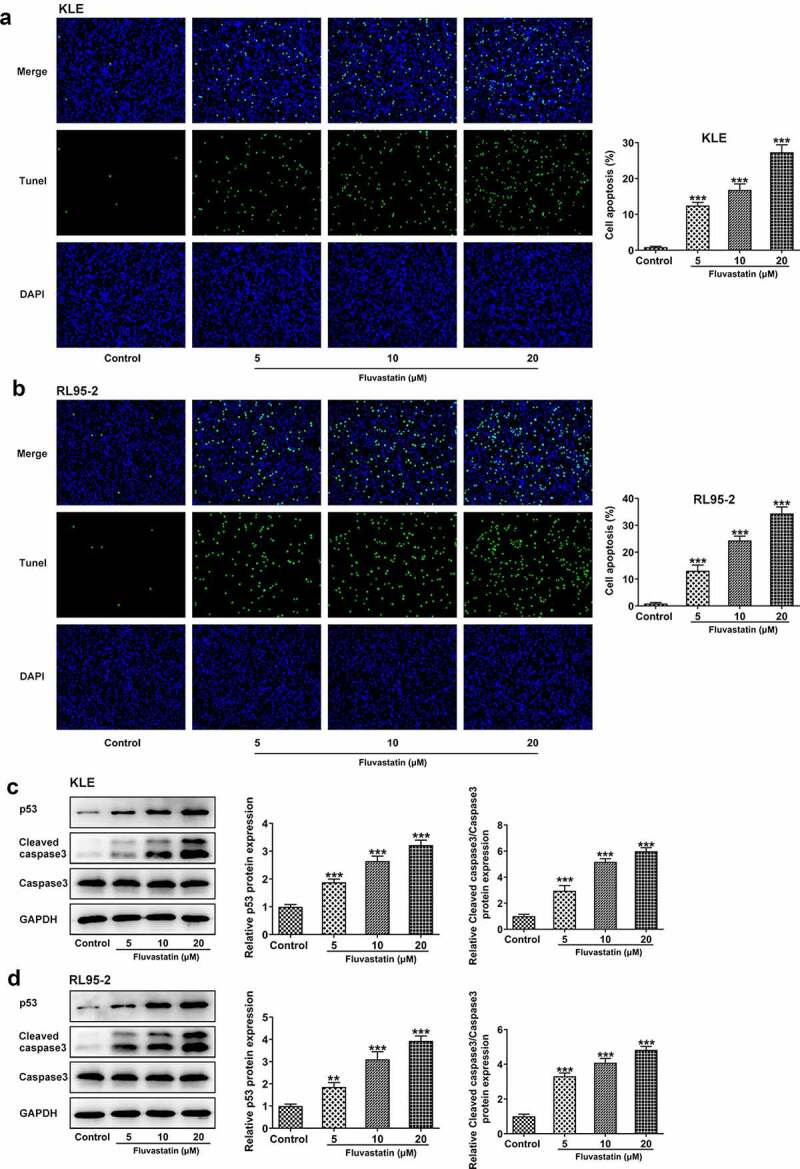
Fluvastatin induces the apoptosis of EC cells. (a-b) The apoptosis and (c-d) apoptosis-related protein expressions in EC cells exposed to fluvastatin. **P < 0.01, *** P < 0.001 versus control

To further determine the effects of fluvastatin on EC cell apoptosis, TUNEL assay was conducted to observe if there were any changes on fluvastatin-exposed EC cells Notably, fluvastatin exposure stimulated the apoptosis of KLE and RL95-2 cells. Tumor suppressor gene P53 and cleaved caspase3 [13,14] are reliable markers for cell apoptosis; therefore, we also employed Western blot to measure their expressions. It was observed that the expressions of p53 and cleaved caspase3 were increased when increasing doses of fluvastatin were added into KLE and RL95-2 cells (Figure 3c-d). Taken together, fluvastatin induces the apoptosis of EC cells.

### Predominant expression of SIRT6 in fluvastatin-exposed EC cells

**Figure 4.**
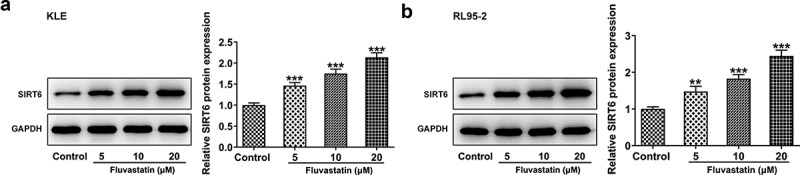
Predominant expression of SIRT6 in EC cells exposed to fluvastatin. (a-b) The expression of SIRT6 in EC cells exposed to fluvastatin. **P < 0.01, ***P < 0.001 versus control

Since previous findings suggested that fluvastatin activated the expression of SIRT6, further cellular experiments were carried out to see whether this finding could be tenable in EC cells. As expected, the data demonstrated that SIRT6 gained a huge growth in fluvastatin-exposed KLE and RL95-2 cells (Figure 4a-b). Moreover, it is noted that fluvastatin induced the expression of SIRT6 in EC cells in a concentration-dependent manner.

### Fluvastatin suppresses the proliferation, migration, and invasion but promotes the apoptosis of EC cells by activating SIRT6

**Figure 5.**
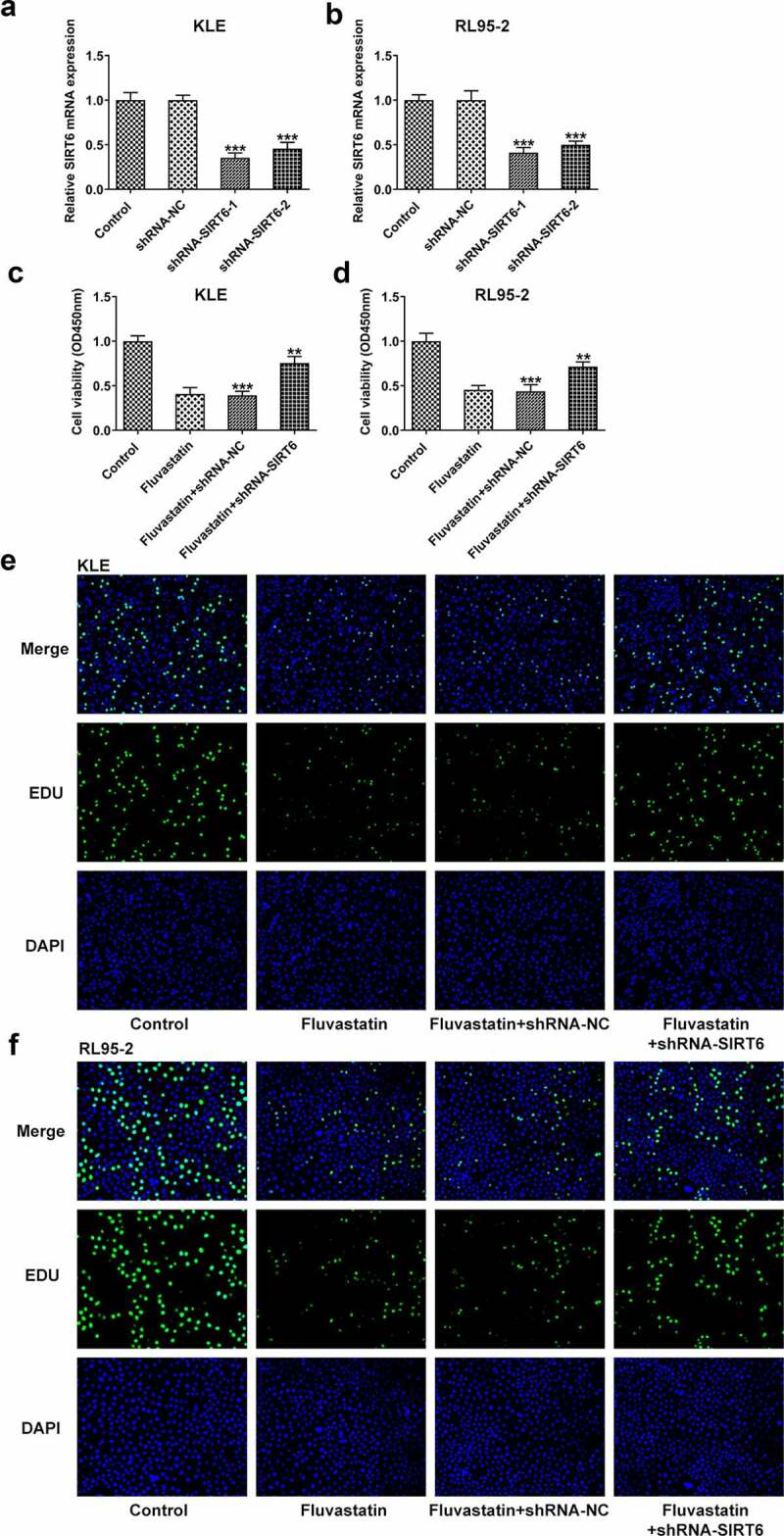
Fluvastatin suppresses the proliferation of EC cells by activating SIRT6. (a-b) The expression of SIRT6 after SIRT6 silencing. (c-d) The cell viability of transfected EC cells exposed to fluvastatin. (e-f) The proliferation of transfected EC cells exposed to fluvastatin. ***P < 0.001 versus control. ^##^P < 0.001 versus fluvastatin + shRNA-NC

**Figure 6.**
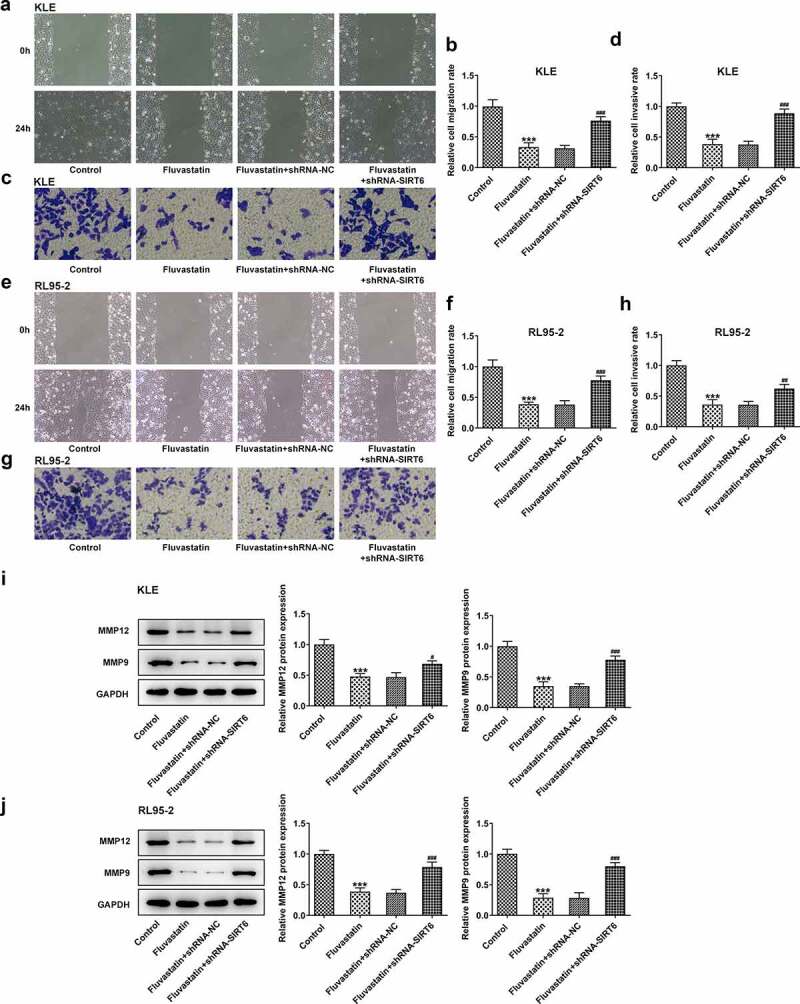
Fluvastatin suppresses the migration and invasion of EC cells by activating SIRT6. (a-b) The migration, (c-d) invasion, (e-f) The migration, (g-h) invasion, and (i-j) MMP12 and MMP9 expression in transfected EC cells exposed to fluvastatin. ***P < 0.001 versus control. ^#^P < 0.05, ^##^P < 0.01, ^###^P < 0.001 versus fluvastatin + shRNA-NC

**Figure 7.**
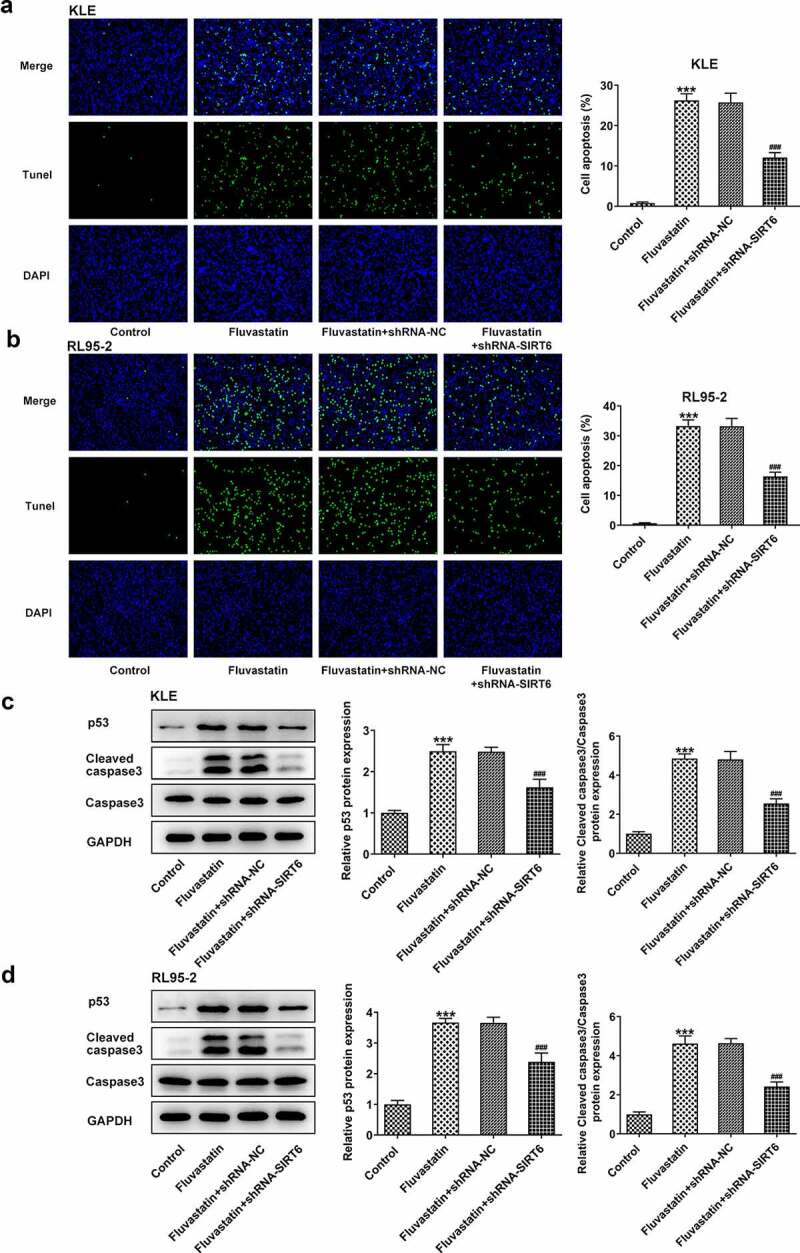
Fluvastatin promotes the apoptosis of EC cells by activating SIRT6. (a-b) The apoptosis and (c-d) apoptosis-related protein expressions in transfected EC cells exposed to fluvastatin. ***P < 0.001 Versus Control. ^###^P < 0.001 versus fluvastatin + shRNA-NC

Next, we examined whether fluvastatin could affect the cellular functions of EC cells by activating SIRT6 expression. After interfering the expression of SIRT6 with corresponding plasmids, ShRNA-SIRT6-1 was chosen for the subsequent assays as it displayed lower expression in EC cells than ShRNA-SIRT6-2 (Figure 5a-b). Results from CCK-8 and EDU implied that the suppressive effects of fluvastatin on the proliferation of EC cells were reversed by SIRT6 silence (Figure 5c-f). Furthermore, SIRT6 silence also enhanced the migration and invasion of EC cells with fluvastatin treatment (Figure 6a-j. Furthermore, the increased apoptosis in fluvastatin-treated KLE and RL95-2 cells was then inhibited by SIRT6 silence, revealing the inhibitory effects of SIRT6 silence on the apoptosis of EC cells with fluvastatin treatment (Figure 7a-d).

## Discussion

Fluvastatin, generally recognized as one of the HMGCR inhibitors, is utilized for the treatment of hypercholesterolemia patients [15]. Fluvastatin has been shown to exhibit anti-tumor activities. The anti-cancer property of fluvastatin was comprehensively illustrated in renal cancer cells [16]. By combining with vemurafenib, fluvastatin can potently exert anti-tumor effects on drug-resistant melanoma cells [17]. In the present study, we found that its anti-tumor activity also worked on EC, because the progressive malignant behaviors of KLE and RL95-2 cells were suppressed while their apoptosis was promoted upon fluvastatin exposure. Of note, previous studies pointed out the side effects that fluvastatin brought about on the body weight of lung cancer mice [18]. Nevertheless, fluvastatin was still deemed as one of the most potent drugs for lung cancer management due to its advantages in inhibition of cancer progression over other HMGCR inhibitors.

SIRT6 isa nuclear protein, which can regulate various cellular pathways, including transcriptional control, metabolism, DNA repair and genomic stability, proliferation and differentiation, cancer and more [19]. It has been reported that SIRT6 serves as either a tumor suppressor gene or an oncogene as it can inhibit tumor formation by improving genomic stability and induce tumor progression by promoting genomic instability [20,21]. Intriguingly, several experts noted the potential of SIRT6 to suppress EC due to the fact that SIRT6 can block the frequent activation of PI3K-mTOR pathway in EC [22]. It has already been reported that fluvastatin, as a drug target can activate the expression of SIRT6, which was confirmed with the results in our study [10]. Then, we further probed whether fluvastatin exerted protective effects on EC progression via knocking down SIRT6 expression. However, the role of PI3K-mTOR pathway in the mechanism of action of fluvastatin related to SIRT6 still requires further study [23,24]. It was obviously found that depletion of SIRT6 triggered more activated cell viability and stimulated the behaviors of EC cells, including proliferation, migration, invasion. Similarly, the proliferation and cloning effects of hepatocellular carcinoma cells were weakened after SIRT6 was knocked out [25].

Consistently, apoptosis of cancer cells was a critical factor influencing the progression of multiple cancers. Accumulating evidence has emerged to suggest that SIRT6 can boost the apoptosis of diverse cancer cells. SIRT6 promoted the apoptosis of nasopharyngeal carcinoma cell lines as Bcl expression was decreased, accompanied by increased TUNEL-positive cells [26]. SIRT6 overexpression also induced apoptosis of gastric cancer cells via regulating JAK2/STAT3 signaling [27]. Consistent with previous studies, we found that silencing SIRT6 led to the decreased apoptosis of KLE and RL95-2 cells, as exhibited by TUNEL. Moreover, Western blot assays revealed that fluvastatin promoted the levels of p53 and cleaved caspase 3 by upregulating SIRT6. We predict that SIRT6 involved in the inhibitory effects of fluvastatin on apoptosis could be related to the regulation for the levels of p53 and cleaved caspase 3. SIRT6 overexpression has been found to induce apoptosis by p53 in a research about cancer [28]. Additionally, there is a report also demonstrating increased expression of SIRT6 promotes apoptosis of endometrial cancer cells by survivin [11]. However, there came different opinions on the role of SIRT6 in apoptosis of cancer cells. For example, SIRT6 suppressed cancer stem-like capacity in tumors with PI3K activation independent of its deacetylase activity. The controversy can be explained by the postulation that SIRT6 may act in a tissue-dependent manner. Simvastatin, which also is a HMGCR inhibitor, possesses a potential anti-tumor effect in endometrial cancer [29,30]. In clinic, fluvastatin had a lower incidence of side effects than Simvastatin in lipid-decreasing. The present study firstly shows the anti-tumor potential of fluvastatin in endometrial cancer and reveals the role of SIRT6 in the mechanism of action of fluvastatin, providing a novel sight for the therapy of endometrial cancer and the study of mechanism of endometrial cancer.

## Conclusion

To sum up, the present study firstly evidenced that fluvastatin suppresses the proliferation, invasion and migration but promotes the apoptosis of endometrial cancer cells by regulating SIRT6 expression. To confirm the accuracy and credibility of our experimental results, we chose two kinds of EC cell lines for experiments. It is the limit of this study for the role of upstream signaling pathway of SIRT6 in the mechanism of action of Fluvastatin in endometrial cancer cells. Further studies on these results should be conducted and the potency and efficiency of fluvastatin should be further validated.

## Data Availability

The datasets used and/or analyzed during the current study are available from the corresponding author on reasonable request.

## Highlights

- Fluvastatin is able to inhibit proliferation, invasion and migration of EC cells.
- SIRT6 mediates the anti-proliferative and anti-metastatic effects of fluvastatin in EC.
- The pro-apoptosis effects of fluvastatin partly were related to p53 and cleaved caspase3.
